# Adipocyte-Targeted Nanocomplex with Synergistic Photothermal and Pharmacological Effects for Combating Obesity and Related Metabolic Syndromes

**DOI:** 10.3390/nano14161363

**Published:** 2024-08-19

**Authors:** Yuanyuan Zhang, Xiaojiao Zeng, Fan Wu, Xiaopeng Yang, Tingting Che, Yin Zheng, Jie Li, Yufei Zhang, Xinge Zhang, Zhongming Wu

**Affiliations:** 1 NHC Key Laboratory of Hormones and Development, Chu Hsien-I Memorial Hospital and Tianjin Institute of Endocrinology, Tianjin Medical University, Tianjin 300134, China; 2 Tianjin Key Laboratory of Metabolic Diseases, Tianjin Medical University, Tianjin 300134, China; 3 Department of Endocrinology, Health Management Center, Tianjin Union Medical Center, Nankai University Affiliated Hospital, Tianjin 300131, China; 4 Key Laboratory of Endocrine Glucose & Lipids Metabolism and Brain Aging, Ministry of Education, Department of Endocrinology, Shandong Provincial Hospital Affiliated to Shandong First Medical University, Jinan 250021, China; 5 Shandong Institute of Endocrine and Metabolic Diseases, Jinan 250021, China; 6 Key Laboratory of Functional Polymer Materials of Ministry Education, Institute of Polymer Chemistry, College of Chemistry, Nankai University, Tianjin 300071, China

**Keywords:** targeted nanocomplex, adipocyte, photothermal therapy, WAT browning, obesity, metabolic syndrome

## Abstract

Obesity is a global epidemic which induces a multitude of metabolic disorders. Browning of white adipose tissue (WAT) has emerged as a promising therapeutic strategy for promoting weight loss and improving associated metabolic syndromes in people with obesity. However, current methods of inducing white adipose tissue browning have limited applicability. We developed a nanocomplex pTSL@(P+I), which is a temperature-sensitive liposome (TSL) surface-conjugated with an adipocyte-targeting peptide (p) and loaded with both browning-promoting agents (P) and photosensitizing agents (I). This nanocomplex exhibits adipocyte targeting, as well as synergistic pharmacological and photothermal properties to promote browning. pTSL@(P+I) effectively upregulates UCP1 and COX5B expression by activating the transcription axis of PPARγ/PGC1α and HSF1/PGC1α, thereby promoting white adipose tissue browning and reducing obesity. This novel nanocomplex exhibited a uniform spherical shape, with an average diameter of approximately 200 nm. Additionally, the nanocomplexes exhibited remarkable photothermal properties and biocompatibility. Further, when adipocytes were treated with pTSL@(P+I), their triglyceride content decreased remarkably and intracellular mitochondrial activity increased significantly. When applied to diet-induced obesity (DIO) mice, the nanocomplex exhibited significant efficacy, demonstrating a notable 14.4% reduction in body weight from the initial measurement, a decreased fat/lean mass ratio of 20.8%, and no statistically significant disparities (*p* > 0.05) in associated side effects when compared to the control group. In summary, implementation of the targeted nanocomplex pTSL@(P+I) to enhance energy expenditure by stimulating white adipose tissue browning offers a promising therapeutic approach for the treatment of obesity and related metabolic syndromes.

## 1. Introduction

Obesity prevalence has tripled over the past half-century, escalating this condition into a global epidemic. By 2025, more than 2.5 billion adults are projected to be affected [1,2]. Obesity is characterized by excessive accumulation of adipose tissue and is frequently accompanied by glycolipid metabolism disorders, which can contribute to the development of chronic metabolic diseases such as diabetes, fatty liver disease, hyperlipidemia, cardio-cerebrovascular disease, and certain cancers [3,4,5,6]. Current approaches to managing obesity primarily include dietary calorie restriction, physical activity, administration of anti-obesity medications, gastric reduction procedures, and liposuction [7,8,9]. These treatment options mainly focus on reducing energy intake [10,11]; however, their efficacy in achieving sustainable weight loss is limited owing to rebound effects after drug withdrawal and potential safety risks [12,13,14].

Recently, promoting energy expenditure to combat obesity has captured the attention of researchers [15,16,17]. Adipose tissue plays a crucial role in energy homeostasis [18], which can be classified into white adipose tissue (WAT) and brown adipose tissue (BAT) based on its anatomical location and metabolic function. Compared with white adipose tissue, BAT cells contain more small lipid droplets and mitochondria that express high levels of the uncoupling protein 1 (UCP1), located in the inner mitochondrial membrane. Uncoupling protein 1 induces an idle state during oxidative phosphorylation by disrupting the coupling between electron transfer and phosphorylation in the respiratory chain. This leads to the dissipation of excess energy through increased heat production [19] and the increased energy usage is beneficial for people with obesity. Recent reports of brown-like adipocytes in the white adipose tissue deposits of adult humans has sparked interest in the usage of thermogenic activity for obesity treatment [20], leading to the exploration of white adipose tissue browning as a target for obesity treatment [21,22]. Hence, strategies that can stimulate the uncoupling of respiration rate and thermogenesis through the transformation of white adipose tissue to BAT could provide alternative approaches for the treatment of obesity and related metabolic syndromes [23,24]. Current methods for inducing white adipose tissue browning can be categorized into two approaches: physiological stimuli and pharmacological treatments [25,26,27]. Notably, traditional strategies relying on physiological stimulation have sub-optimal efficiency with unsatisfactory stability [28,29,30], whereas non-local stimulation may cause scalding or frostbite in peripheral healthy tissues. Moreover, systemic administration of pharmacological treatments can lead to varying degrees of gastrointestinal, hepatic, and renal damage. Such treatments may even cause adverse reactions affecting the function of the heart or central nervous system; consequently, their use in people with obesity is limited. Therefore, research into novel methods of inducing white adipose tissue browning are urgently required.

**Scheme 1:**
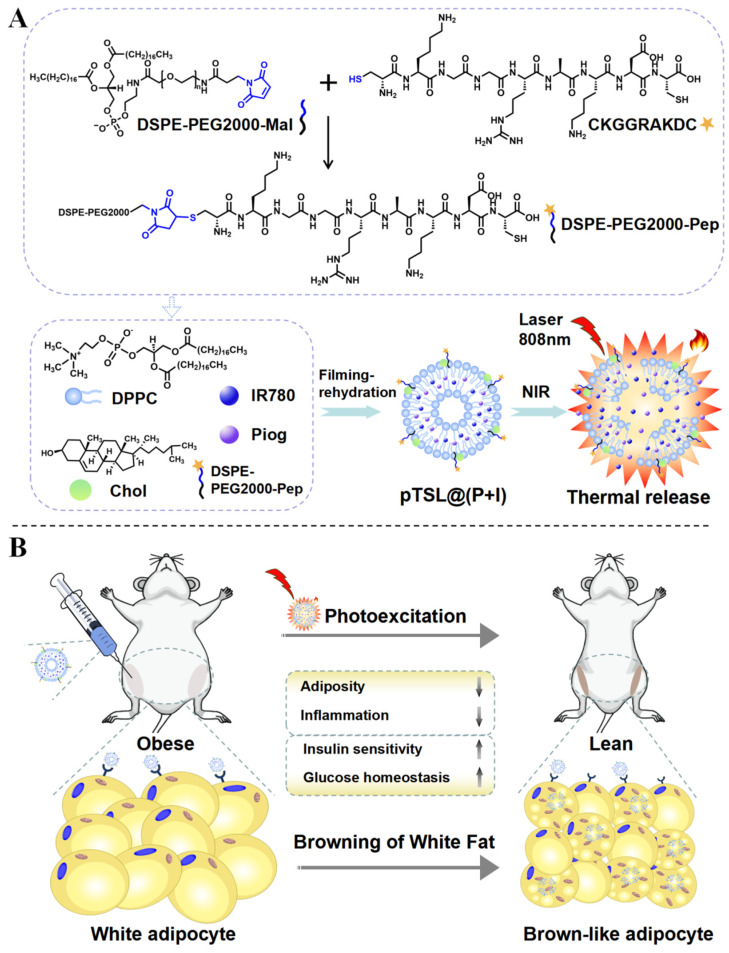
(**A**) Composition and structure of targeted nanocomplex pTSL@(P+I). (**B**) NIR light-activated targeted nanocomplex for white adipose tissue browning to enhance anti-obesity effects in mice.

Herein, we developed an adipocyte-targeted nanocomplex, pTSL@(P+I), with synergistic pharmacological and photothermal properties. This nanocomplex effectively upregulates UCP1 and COX5B expression by activating the transcription axis of PPARγ/PGC1α and HSF1/PGC1α, thereby promoting white adipose tissue browning and improving obesity (Scheme 1A). Specifically, the peptide motif (CKGGRAKDC sequence) was inserted on the surface of the nanocomplex to specifically bind prohibitin, which is highly expressed in the adipose vascular system and differentiated adipocytes [31,32]. The nanocomplex was injected subcutaneously into mouse white adipose tissue to enhance adipocyte uptake through specific binding to prohibitin (Scheme 1B). The nanocomplex was subsequently stimulated by 808-nm near-infrared (NIR) light to generate a photothermal effect that resulted in localized hyperthermia, which promoted the cleavage of nanocomplexes and efficient release of medication at the local site. Considering the synergistic action of its photothermal and pharmacological effects, the nanocomplex can effectively promote white adipose tissue browning locally, increase energy expenditure, and improve obesity. This study therefore provides a novel therapeutic strategy for combating obesity and related metabolic syndromes.

## 2. Materials and Methods

### 2.1. Materials

Dipalmitoyl stearoylcholine (DPPC) was purchased from TCI (Shanghai, China). Cholesterol (Chol) was purchased from Heowns (Tianjin, China). Phospholipid polyethylene glycol 2000 maleimide (DSPE-PEG2000-Mal) was purchased from Xi’an Ruixi Biotechnology Co., Ltd. (Xi’an, China). Targeted peptide (CKGGRAKDC) was purchased from Gill Biochemicals Ltd. (Shanghai, China). IR780 iodide was purchased from Shanghai Sarn Chemical Technology Co. (Shanghai, China). Pioglitazone (Piog) hydrochloride, dexamethasone (Dex), and penicillin-streptomycin antibiotics were purchased from Beijing Solarbio Technology Co., Ltd. (Beijing, China). Insulin and 3-isobutyl-1-methylxanthine (IBMX) were purchased from Shanghai Biyuntian Biotechnology Co., Ltd. (Shanghai, China). DMEM high-glucose medium, fetal bovine serum (FBS), and trypsin-containing digest with EDTA were purchased from Gibco (New York, NY, USA). All other reagents and chemicals were of analytical grade.

### 2.2. Synthesis of Peptide-Modified DSPE-PEG2kDa-Mal

The peptide (CKGGRAKDC) targeting prohibitin was conjugated to DSPE-PEG2000-Mal as described previously [33], and the product was labeled as DSPE-PEG2000-Pep. Briefly, CKGGRAKDC and DSPE-PEG2000-Mal were dissolved in distilled water. The CKGGRAKDC solution (10 mM) and DSPE-PEG2000-Mal solution (10 mM) were mixed in the ratio of 1:1 (*w*/*w*), and the reaction was continuously stirred at 4 °C for 12 h. The reaction was lyophilized after 24 h of dialysis. The ^1^H NMR spectra of DSPE-PEG2000-Mal (with CDCl_3_ as solvent) and DSPE-PEG2000-Pep (with CD_4_O as solvent) were determined by nuclear magnetic resonance spectrometer (AVANCE III 400 MHz, Bruker, Karlsruhe, Germany) at 25 °C, respectively.

### 2.3. Construction and Characterization of the Nanocomplex pTSL@(P+I)

Blank nanocomplex was prepared by fixing the molar ratio of DPPC:DSPE-PEG2000-Pep:Chol to 16:1:4 by using thin-film hydration. In order to determine the optimal feeding ratio of Piog and IR780, we fixed the feeding mass ratio of IR780 to nanocomplex as 11%, then three kinds of targeted nanocomplex pTSL@(P+I) was prepared by changing the feeding mass ratio of Piog to nanocomplex (18%, 22%, and 26%, *w*/*w*) [34]. DPPC, DSPE-PEG2000-Pep, Chol, IR780, and Piog were dissolved in a chloroform/methanol solvent mixture (3:2, *v*/*v*), and the solutions were rotary evaporated at 40 °C to obtain lipid films. Subsequently, the lipid film was hydrated using PBS in a round-bottomed flask by rotary shaking at 180 rpm at a hydration temperature of 60 °C until a single dispersed phase emulsion was formed. The emulsion was ultrasonically shaken and then passed through a 200 nm microporous filter membrane five times to obtain pTSL@(P+I) of uniform size. pTSL@P loaded with Piog, pTSL@I loaded with IR780, and TSL@(P+I) unspliced with peptides were prepared in the same way.

The particle size and ζ potential of pTSL@(P+I) were evaluated by dynamic light scattering (DLS). The morphology of pTSL@(P+I) was observed by transmission electron microscope (TEM). The UV–visible absorption spectrum of pTSL@(P+I) suspension was scanned and recorded using a UV spectrophotometer (Shimadzu 2250, Shimadzu, Kyoto, Japan)). To test the stability of pTSL@(P+I), it was placed in 25 °C and 37 °C environments, respectively, and the changes in size of pTSL@(P+I) were monitored by DLS at specified time points over 72 h.

The loading capacity (LC) was determined by UV–visible spectrophotometry. Briefly, the pTSL@(P+I) suspension was centrifuged at 4 °C (13,000 rpm, 30 min), and the supernatant was removed. The precipitated pTSL@(P+I) was re-dispersed in PBS solution, and the absorbance values of the suspension at 269 nm (Piog) or 780 nm (IR780) were measured by UV–visible spectrophotometry after methanol demulsification. Then, the loading capacity of pTSL@(P+I) for Piog and IR780 was calculated separately based on the standard curves of Piog and IR780.

### 2.4. Photothermal Property of pTSL@(P+I)

To evaluate the photothermal properties of pTSL@(P+I), a 808 nm NIR laser (1 W/cm^2^) was delivered through a centrifuge tube at different concentrations (62.5, 125, 250, 500, and 1000 μg/mL). The temperature change was recorded once every minute by an infrared thermal imager (FLIR E50, FLIR, Portland, OR, USA).

### 2.5. In Vitro Drug Release from pTSL@(P+I)

To evaluate the effect of temperature on drug release form pTSL@(P+I), 5 mL (1 mg/mL) of pTSL@(P+I) suspension was transferred into a dialysis bag (weight cutoff, 3.5 kDa) and the bag was placed in a centrifuge tube with 15 mL PBS buffer. The drug release was carried out under a 37 °C and 45 °C thermostatic oscillator, respectively. At predetermined time (0, 2, 4, 8, 12, 24, 36, 48, 60, and 72 h), 200 μL of buffer was extracted and supplemented with the same volume of fresh PBS. The absorbance values of the extracts at 269 nm were measured by a UV–visible spectrophotometer and the Piog content was calculated based on the drug’s standard curve.

### 2.6. Biocompatibility of pTSL@(P+I)

Fresh human blood was used as the sample of the hemolysis test. The blood was centrifuged at 4000 rpm for 5 min, and the supernatant was removed. The red blood cells were collected and rinsed with PBS three times, and then dispersed in PBS for storage. Subsequently, we mixed 0.5 mL of PBS solution of samples (62.5, 125, 250, 500, and 1000 μg/mL) with 0.5 mL of red blood cell (2%) suspension. The mixed solution was then cultured at 37 °C for 1 h, and the absorbance of the supernatant at 576 nm was measured with a microplate reader after centrifugation. One percent Triton X-100 solution was used as the positive control, and PBS was used as the negative control.

Hemolytic rate (%) = (A_Sample_ − A_PBS_)/(A_Triton X-100_ − A_PBS_) × 100%

where A_Sample_ is the absorbance of samples treated red blood cells, A_PBS_ is the absorbance of red blood cells in negative control group, and A_Triton X-100_ is the absorbance of cells in positive control group. Parallel experiments were performed 3 times to reduce the experimental error.

The toxicity of pTSL@(P+I) against 3T3-L1 cells and differentiated 3T3-L1 cells was determined using the CCK-8 (Solarbio CA1210, Solarbio, Beijing, China) assay. The nanocomplex was diluted with medium to achieve a predetermined series of double dilution concentrations (62.5, 125, 250, 500, and 1000 μg/mL). The two types of 3T3-L1 cells were inoculated at 1 × 10^4^ in 96-well plates and further incubated at 37 °C in 5% CO_2_/95% air for 24 h. Then, the medium was replaced with the pTSL@(P+I) solution as formulated above. Following further incubation for 48 h, the CCK-8 solution was added to each well and incubated for another 1 h. A microplate reader was used to measure the absorbance at 450 nm.

### 2.7. In Vitro Cellular Uptake of pTSL@(P+I)

Differentiated 3T3-L1 cells were inoculated in 6-well plates at a density of 1 × 10^5^ cells/well and incubated at 37 °C for 24 h. Then, the medium was replaced with fresh medium containing FITC-labeled TSL@(P+I) or pTSL@(P+I). Meanwhile, to verify the specific recognition of 3T3-L1 cells by the targeted peptide of pTSL@(P+I), anti-prohibitin monoclonal antibody was added to pre-incubate for 30 min prior to the addition of pTSL@(P+I). After co-incubation for 1 and 3 h, respectively, the cells were stained with DAPI to locate the nucleus. Finally, the cells were observed and photographed by laser confocal microscopy (CLSM, TCS SP8, Leica, Weztlar, Germany). Fluorescence quantification analysis was performed using Image J 1.8.0 software.

### 2.8. In Vitro Pro-Browning Ability of pTSL@(P+I)

We cultured 3T3-L1 cells in high-glucose DMEM containing 10% (*v*/*v*) fetal bovine serum and 1 × penicillin-streptomycin until cell confluence. Differentiation was induced by the classical cocktail induction method (MDI) [35]. Briefly, differentiation was induced by treating the cells with a DMEM lipid-forming medium containing 10 μg/mL insulin, 1 μM Dex, and 0.5 mM IBMX from day 0 to day 2. Subsequently, a DMEM lipid-forming medium containing only 10 μg/mL insulin was used from day 3 to day 7. The materials (Piog, pTSL@P, pTSL@I, or pTSL@(P+I) were added from day 5 to day 7 for intervention. The concentration content of Piog was 50 μg/mL in all cases, the concentration content of IR780 was 30 μg/mL in all cases, and in pTSL@I and pTSL@(P+I) intervention groups we applied 808 nm laser to irradiate cells for 5 min every day. At the same time, PBS intervention was set as the control group.

Lipid accumulation was assessed by Oil Red O (ORO) staining. Cells were fixed with ORO Fixative solution for 30 min. After washing with PBS, cells were immersed in 60% isopropyl alcohol for 5 min, and stained with freshly prepared ORO Stain for 15 min. After removing the unstained dye by PBS washing, isopropanol was added to extract the dye within the cells, and the absorbance at 490 nm was measured to quantify the lipid accumulation.

Adipocytes after intervention were crushed with an ultrasonic cell crusher (SONICS, VCX150, SONICS, Newtown, CT, USA), and triglyceride (TAG) and free fatty acid (FFA) levels were measured using a triglyceride or free fatty acid content determination kit (Nanjing Jiancheng Bioengineering Institute) according to the manufacturer’s protocol.

Mito-Tracker Red CMXRos stock solution (200 μM) was obtained by dissolving 50 μg of Mito-Tracker Red CMXRos powder in 470 μL of anhydrous DMSO. The staining solution was prepared by diluting the stock solution with culture medium (1:1000) and incubating at 37 °C. Then, the medium of the adipocytes was replaced with the prepared staining solution, which was incubated at 37 °C for 25 min. Finally, the adipocytes were fixed with 4% paraformaldehyde and the nuclei were stained with DAPI. Fluorescence imaging was observed and photographed on the CLSM.

### 2.9. Evaluation of In Vivo Anti-Obesity Ability of pTSL@(P+I)

Male C57BL/6 mice (6 weeks old) were purchased from SiPeiFu Biotechnology Co., Ltd. (Beijing, China), and all animal studies were conducted in accordance with the NIH Animal Care Guidelines and approved by the Ethics Committee of Tianjin Medical University (SYXK(J):2020-0001). The animals were housed in a standard conditioned animal house (temperature: 22–24 °C; humidity: 60%; light/dark cycle: 12 h/12 h; free feeding and drinking), and the bedding was changed regularly every day. The dietary treatment was started when the mice were 7 weeks old. The mice were divided into a normal diet control (Ctrl) group and a high-fat diet (HFD, D12492, purchased from Sainuo Biotechnology Co., Ltd., Jilin, China, fat content accounts for 60% of total calories) induced obesity (DIO) model group. After about 8 weeks, the diet-induced obesity model was finished when the weight of the mice of HFD group exceeded 20% of that of Ctrl group. The diet-induced obesity mice were randomly divided into 5 groups (*n* = 5): PBS group, Piog group, pTSL@P group, pTSL@I group, and pTSL@(P+I) group, and were respectively given bilateral subcutaneous injections into inguinal white adipose tissue (iWAT) with PBS, Piog (Piog content: 1 mg/kg), pTSL@P (Piog content:1 mg/kg), pTSL@I (IR780 content: 0.6 mg/kg, irradiated with 0.5 W/cm^2^ 808 nm NIR laser for 5 min) and pTSL@(P+I) (Piog content: 1 mg/kg; IR780 content: 0.6 mg/kg, irradiated with 0.5 W/cm^2^ 808 nm NIR laser for 5 min). The treatments were administered once every two days for 28 days. During the treatment period, food intake and body weight changes were recorded every three days. At the end of the treatment, the mice were executed, blood was extracted from the medial canthus, and the relevant tissues were collected, weighed, and photographed.

To evaluate the in vivo photothermal effect of pTSL@(P+I), we gave each group of mice a 0.5 W/cm^2^ 808 nm NIR laser to irradiate the left groin, and infrared thermography was performed on the mice with an infrared thermography camera (FLIR E50) at intervals of 1 min, for a total of 5 min. The pseudo-color images and the temperatures were quantitatively analyzed with FLIR tools.

To investigate the in vivo biodistribution of pTSL@(P+I), FITC-labeled pTSL@(P+I) was prepared using the aforementioned method. Mice were subcutaneously injected with 0.5 mg/mL FITC-labeled pTSL@(P+I) suspended in PBS into the left inguinal white adipose tissue. Images were taken with the In-Vivo Imaging System (Kodak, Rochester, NY, USA) at 1, 24, and 48 h post-injection. Then, the mice were euthanized at 24 and 48 h post-injection, respectively. Their major organs (heart, liver, kidney, lung, spleen) and left iWAT were isolated. The biodistribution of FITC-labeled pTSL@(P+I) in the major organs and iWAT of mice at 24 and 48 h post-injection was also assessed.

Glucose tolerance tests (GTT) and insulin tolerance tests (ITT) were performed before the end of the treatment, respectively. Glucose tolerance testing was performed after 12 h of overnight fasting in mice with rapid intraperitoneal injection of glucose at a dose of 2 g/kg, and insulin tolerance test was performed after 6 h of fasting in mice with intraperitoneal injection of insulin at a dose of 0.75 U/kg. The glucose concentration in mice tail veins was measured using a Sinocare blood glucose meter (GA-3) at 0, 15, 30, 60, 90, and 120 min after the injection, respectively.

Before the end of the animal experiments, the small animal body fat analyzer NMR Analyzer mq 7.5 (BRUKER, Karlsruhe, Germany) was applied to detect and record the weight of fat and lean tissue of each mouse according to the manufacturer’s protocol.

### 2.10. Measurement of Animal Energy Metabolism

Metabolic parameters were determined for each mouse using PROMETHION BX1 metabolic cages (SABLE SYSTEMS INTERNATIONAL, Las Vegas, NV, USA) before the end of the animal experiments. The body weights of representative mice from each group were recorded before entering the physiological cage. After several hours of acclimatization, the number of autonomic activities, oxygen (O_2_) consumption, carbon dioxide (CO_2_) production, and respiratory quotient (RQ) of the mice were monitored and recorded over a 24 h period. The mice were kept in a light/dark cycle for 12 h at 22 °C and fed and watered ad libitum.

For cold tolerance testing, diet-induced obesity mice were placed in a 4 °C freezer for 4 h with free access to food and water [36,37]. Rectal temperatures were measured at specified time points (0, 0.5, 1, 2, and 4 h).

### 2.11. Histological Assessment

After mice were executed at the end of the experiment, adipose tissues (inguinal white adipose tissue, i.e., iWAT, epididymal white adipose tissue i.e., eWAT, and interscapular BAT) and major organs (liver, kidney, and heart) were excised, weighed, and fixed in 4% paraformaldehyde solution. The fixed tissues were dehydrated with gradient ethanol, embedded in paraffin blocks, and sectioned with a paraffin slicer, then analyzed by hematoxylin and eosin (H&E) staining and photographed for observation under a light microscope. The tissue sections were analyzed by immunohistochemistry with anti-UCP1 antibody (1:500 dilution, Proteintech, Chicago, IL, USA), and photographed under a light microscope. Immunofluorescence staining with anti-UCP1 antibody (1:50 dilution) was visualized using a CLSM.

### 2.12. Western Blot and Inflammatory Factor Analysis

The adipocytes (1 × 10^6^) or iWAT (100 mg) were lysed with RIPA lysis buffer (Solarbio, R0010, Beijing, China) containing PMSF (Solarbio, P0100) and Protein Phosphatase Inhibitor Mix (Solarbio, P1260). The total protein lysates were boiled with a loading buffer containing 10% SDS-PAGE. Subsequently, the isolated proteins were transferred to PVDF membranes. The PVDF membrane blots were closed in 5% skimmed milk at room temperature for 2 h, washed in Tris-buffered saline containing Tween 20, and incubated at 4 °C overnight. The membrane was blotted with rabbit anti-UCP1 (Proteintech, 23673), rabbit anti-PPARγ (Proteintech, 16643), rabbit anti-COX5B (Proteintech, 11418), rabbit anti-HSF1 (Proteintech, 16107), and mouse anti-PGC1α (Proteintech, 66369) primary antibodies. Anti-rabbit IgG (Abclonal, AS014, Wuhan, China) was used as a secondary antibody to UCP1, PPARγ, COX5B, and HSF1, and anti-mouse IgG (Abclonal, AS003) was used as a secondary antibody to PGC1α. Detection was subsequently achieved using ECL chemiluminescent solution, and images were obtained using a chemiluminescent analyzer (Tanon 4600SF, Shanghai, China).

In the in vitro experiment, the adipocyte (2 × 10^5^) culture supernatant after intervention was collected, and for the in vivo experiment, the iWAT (80 mg) was ground, homogenized, and centrifuged to collect the supernatant. IL-1β and TNF-α inflammatory factors were assayed following the instructions of the ELISA kit (ELISA Bio, Shanghai, China, ml301814S for IL-1β, and ml002095S for TNF-α).

### 2.13. Evaluation of Serologic Parameters

After euthanizing the mice, their eyeballs were removed to collect blood, which was left at room temperature for 2 h. Subsequently, the blood was centrifuged at 4 °C and 1500 rpm for 30 min to obtain serum. The levels of triglyceride, total cholesterol (TC), low density lipoprotein cholesterin (LDL-C), total protein (TP), alanine aminotransferase (ALT), aspartate aminotransferase (AST), serum creatinine (SCr), uric acid (UA), urea nitrogen (UREA), creatine kinase (CK), creatine kinase-isoenzyme (CKMB), and lactate dehydrogenase (LDH) were measured using an automatic biochemical analyzer (BS-240VET, Mindray, Shanghai, China).

### 2.14. Statistical Analysis

The experimental data were expressed as mean ± standard deviation with *n* ≥ 3. All data were statistically analyzed using GraphPad Prism 8.0. Comparisons between two groups of data were performed using unpaired *t*-tests, and in comparisons among multiple groups of data, one-way ANOVA was used for one variable and two-way ANOVA was used for more variables. *p* < 0.05 was considered statistically significant for differences between groups. Vector images in the text were processed and stitched using PS (Adobe Photoshop 2020) and AI (Adobe Illustrator 2019) software.

## 3. Results and Discussion

### 3.1. Preparation and Characterization of pTSL@(P+I)

**Figure 1:**
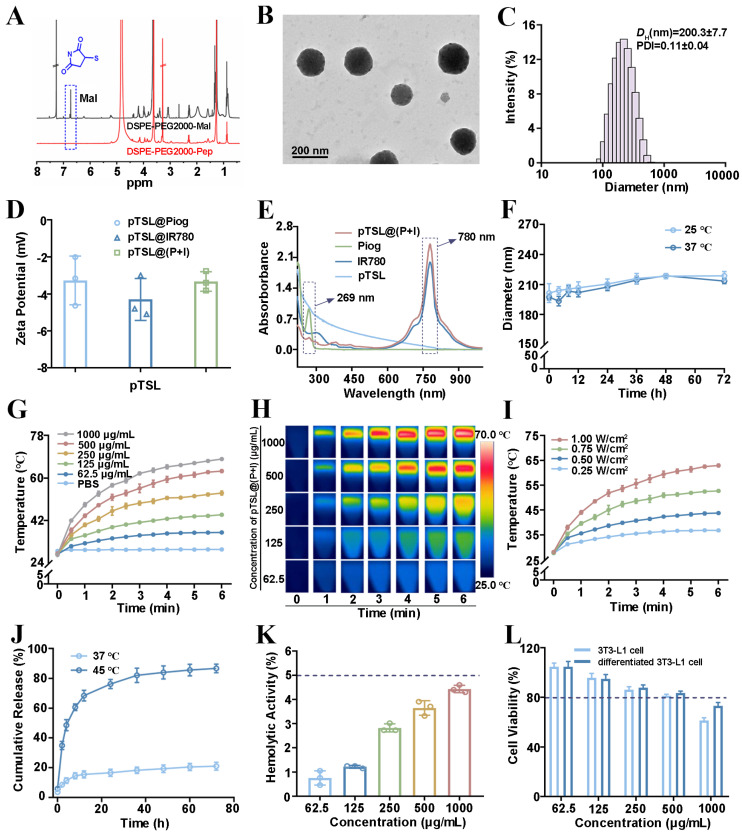
(**A**) ^1^H NMR spectra of DSPE-PEG2000-Mal in CDCl_3_ and DSPE-PEG2000-Pep in CD_4_O. (**B**) TEM images of targeted nanocomplex pTSL@(P+I). (**C**) Histogram of the particle size distribution of pTSL@(P+I) measured by DLS. (**D**) Potential distribution of the targeted nanocomplex measured by DLS. (**E**) UV–visible-near infrared (UV–VIS-NIR) spectrum of pTSL@(P+I). (**F**) Particle size stability of pTSL@(P+I) at 25 °C and 37 °C measured by DLS. Photothermal conversion curves (**G**) and photothermal images (**H**) of different concentrations of pTSL@(P+I) after irradiation with 808-nm NIR light (1.0 W/cm^2^) for various durations. (**I**) Photothermal conversion curves of pTSL@(P+I) at a concentration of 500 μg/mL under varying powers and durations when exposed to 808-nm NIR light. (**J**) Cumulative release curves of Piog from pTSL@(P+I) at 37 °C and 45 °C, respectively. (**K**) Hemolysis of different concentrations of pTSL@(P+I). (**L**) 3T3-L1 cell and differentiated 3T3-L1 cell survival rates measured by CCK-8 method after treatment with different concentrations of pTSL@(P+I) for 48 h.

To effectively and precisely enhance white adipose tissue browning and ultimately ameliorate obesity, we developed a NIR-activated nanocomplex that specifically targets adipocytes. The CKGGRAKDC-modified DSPE-PEG2000-Mal was obtained, labeled as DSPE-PEG2000-Pep, and its structure was confirmed through nuclear magnetic hydrogen spectroscopy (^1^H NMR). As depicted in Figure 1A, the peak at 6.7 ppm represented the characteristic absorption peak of the carbon–carbon double bond on maleimide (Mal), which disappeared after the coupling reaction. This confirmed that DSPE-PEG2000-Pep was successfully synthesized via a sulfhydryl-olefin click chemistry reaction. Three formulations of the targeted nanocomplex, pTSL@(P+I), are referred to as pTSL@(P+I)1, pTSL@(P+I)2, and pTSL@(P+I)3, respectively. They were all prepared via thin-film hydration and the three formulations were created by altering the feeding mass ratio of IR780 to Piog. Notably, pTSL@P, pTSL@I, and TSL@(P+I) were prepared using the same method. Studies have shown that neutral nanocomplexes (within ±10 mV) with a particle size in the range of 100–200 nm were more evenly distributed in tissues [38]. Importantly, the high loading capacity of nanocomplexes ensures efficient treatment at the targeted sites [39,40]. Considering factors such as particle size and drug-loading capacity of these nanocomplexes (Supplementary Table S1), we selected pTSL@(P+I)2 for subsequent experiments. At a concentration of 1000 μg/mL for the nanocomplex, the phospholipid concentration was 583.33 μg/mL, and the targeting peptide concentration was 46.60 μg/mL. The nanocomplex exhibited an average diameter of approximately 200 nm with a uniform spherical shape when observed under TEM (Figure 1B). DLS analysis revealed that the particle size was 200.30 ± 7.70 nm (Figure 1C), while the zeta potential was −3.40 ± 0.43 mV (Figure 1D). Since Piog and IR780 were encapsulated in the nanocomplex, pTSL@(P+I) showed absorption peaks similar to those of Piog (269 nm) and IR780 (780 nm), as shown in Figure 1E. Within 72 h, the particle size (Figure 1F) and dispersion coefficient (Supplementary Figure S1) of pTSL@(P+I) remained stable at both 25 and 37 °C, indicating the excellent stability of pTSL@(P+I) under physiological conditions.

IR780 could be activated to convert the absorbed photons into heat under NIR laser irradiation, and the temperature change in pTSL@(P+I) at different concentrations was recorded. There were positive correlations between the amplitude of the temperature rise and the concentration of nanocomplexes (62.5, 125, 250, 500, and 1000 μg/mL), light power (0.25, 0.50, 0.75, and 1.00 W/cm^2^), as well as light duration (1, 2, 3, 4, 5, and 6 min) (Figure 1G–I). As depicted in Figure 1G,H, when the 808 nm NIR light power was fixed at 1.0 W/cm^2^, the temperature of the nanocomplex increased with increases in both nanocomplex concentration and light exposure time. As shown in Figure 1I, when the nanocomplex concentration was fixed at 500 μg/mL, the temperature of the nanocomplexes could be elevated from 27.7 to 43.2 °C when exposed to a light power intensity of 0.5 W/cm^2^ for 5 min. Importantly, phospholipid DPPC underwent phase transition at approximately 41 °C [41] and hyperthermia (41 ± 0.5 °C) induced thermogenesis and brown gene programs in vitro and vivo [17]. These observations indicate that NIR light energy absorbed by IR780 can be effectively converted into thermal energy for drug release from nanocomplexes and plays an important role in photothermal and pharmacological synergistic therapy.

### 3.2. In Vitro Piog Release from pTSL@(P+I)

Piog is an FDA-approved PPARγ agonist used to treat metabolic diseases, such as Type 2 diabetes mellitus. The benefits of Piog are insulin sensitization, improved lipid metabolism, and regulation of inflammation [42,43]. To assess the efficacy of pTSL@(P+I), we first evaluated the drug release capability of the nanocomplexes. Considering that the phase transition temperature of DPPC is approximately 41 °C [41,44], we evaluated the drug release behavior of pTSL@(P+I) at 37 and 45 °C (Figure 1J). At 37 °C, pTSL@(P+I) exhibited minimal drug leakage, with only a 21% cumulative release of Piog within 72 h. Conversely, at 45 °C, Piog release continued to increase in a heat-dependent manner. The initial Piog release rate from pTSL@(P+I) reached nearly 50% within the first 4 h and ultimately achieved an overall release rate close to 90% within a span of three days. These findings suggest that the flow-type cavities present in the nanocomplexes facilitate rapid drug release. Consequently, it can be inferred that pTSL@(P+I) remained relatively stable under physiological conditions but exhibited accelerated drug release when exposed to temperatures higher than its phase-transition temperature. This property enhances precise drug delivery, thereby specifically targeting the adipose tissue.

### 3.3. Biocompatibility of pTSL@(P+I)

Biocompatibility of nanocomplexes for biomedical applications is a crucial concern. As shown in Figure 1K, the co-incubation of erythrocytes with pTSL@(P+I) solution at various concentrations for 1 h did not result in any significant hemolysis (all hemolysis values were below 5% and complied with the standard [45]). This indicates that pTSL@(P+I) did not exhibit noticeable toxicity towards erythrocytes.

We further assessed the cytotoxicity of the nanocomplex on 3T3-L1 cells and differentiated 3T3-L1 cells using the CCK-8 assay. Following treatment with varying concentrations (62.5, 125, 250, 500, and 1000 μg/mL) of solutions of pTSL@(P+I) for 48 h, the proliferation rates of 3T3-L1 cells and differentiated 3T3-L1 cells were quantified as shown in Figure 1L. Except for the group with the highest concentration of pTSL@(P+I), both groups with other concentrations of intervention had a cell proliferation rate exceeding 80%. These findings demonstrate the excellent biocompatibility of the nanocomplex and highlight its potential for clinical applications.

### 3.4. Efficient Cellular Uptake of pTSL@(P+I)

The specific binding of the peptide motif CKGGRAKDC to prohibitin, a membrane protein highly expressed in the adipose vascular system and differentiated adipocytes, has been previously reported, indicating its association with white adipocytes [31,32,46,47]. To explore its adipocyte-targeting properties, we utilized differentiated adipocytes and FITC-labeled pTSL@(P+I) for further investigation.

**Figure 2:**
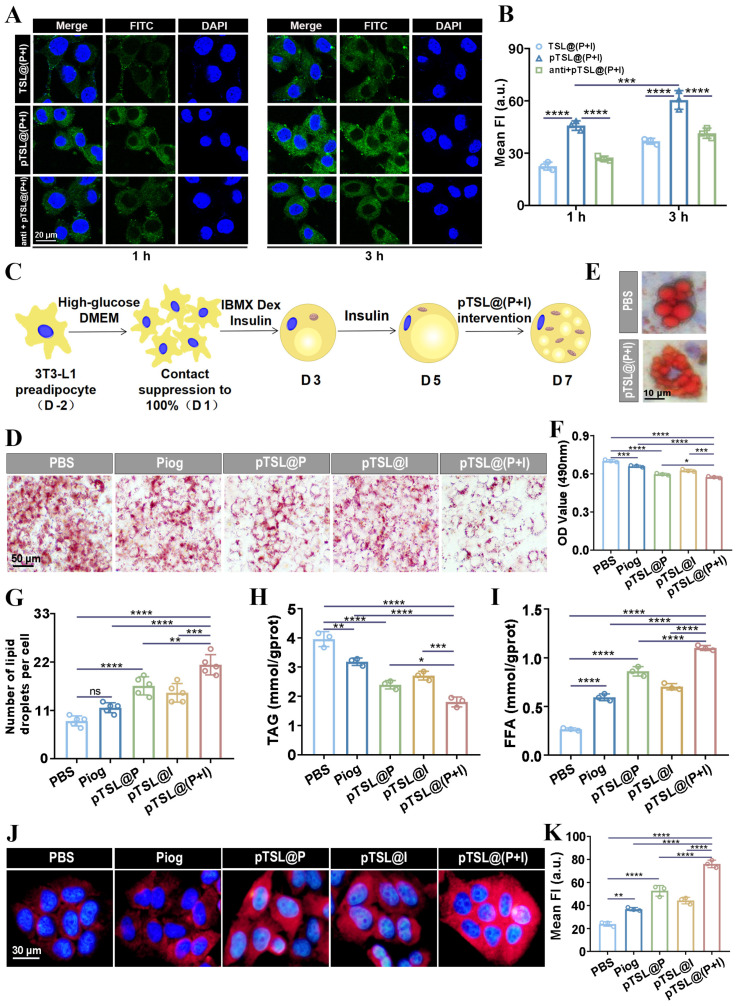
(**A**) After labeling TSL@(P+I) and pTSL@(P+I) with FITC fluorescent probes, CLSM images of differentiated 3T3-L1 cells following co-incubation with TSL@(P+I), pTSL@(P+I), and anti-prohibitin + pTSL@(P+I) for 1 h (left) or 3 h (right), respectively. (the DAPI-stained nucleus as blue fluorescence, the FITC-labeled nanocomplex as green fluorescence) (**B**) Quantification of the average fluorescence intensity (FI) of FITC in 3T3-L1 cells from each experimental group in (**A**) using Image J 1.8.0 software. (**C**) Schematic diagram of cell culture and intervention process. Oil Red O-stained images of 3T3-L1 cells induced differentiation and intervened in each group (**D**,**E**) and quantification of lipid droplets after extraction with isopropanol (**F**). (**G**) Quantification of the number of lipid droplets per cell following various interventions (*n* = 5). Concentrations of lipid triglyceride (TAG) (**H**) and free fatty acid (FFA) (**I**) in differentiated 3T3-L1 cells subjected to various interventions. Visualization of mitochondrial fluorescence (**J**) in differentiated 3T3-L1 cells following various interventions and quantification of the average fluorescence intensity (FI) (**K**) in cells from each experimental group in (**J**) using Image J 1.8.0 software. (the DAPI-stained nucleus as blue fluorescence, the Mito-Tracker as red fluorescence) * *p* < 0.05, ** *p* < 0.01, *** *p* < 0.001, **** *p* < 0.0001; ns = not significant. Statistical significance was assessed via two-way ANOVA (**B**) and one-way ANOVA (**F**–**K**).

The CLSM results in Figure 2A and quantification results based on FITC-based analysis using Image J 1.8.0 software in Figure 2B showed the DAPI-stained nucleus as blue fluorescence, and the FITC-labeled nanocomplex entering the cytoplasm through endocytosis as green fluorescence. The FITC signal intensity of cells in the pTSL@(P+I) group was significantly higher than that of the TSL@(P+I) group, with increases of 102.7% and 64.5% compared to TSL@(P+I), at 1 and 3 h, respectively. Moreover, pre-incubation of 3T3-L1 cells with an anti-prohibitin monoclonal antibody for 0.5 h to block prohibitin receptors on cell surfaces resulted in a reduction in the green fluorescent signal compared to cells without preincubation. Specifically, when pre-cultured with anti-prohibitin monoclonal antibody for 0.5 h before co-incubation with pTSL@(P+I), the FITC signal intensity of cells decreased compared to that of cells without pre-culture, regardless of whether they were co-incubated for 1 or 3 h, with reductions of 41.5% and 31.6%, respectively. These findings confirmed that the inclusion of the prohibitin-targeting peptide enhanced the targeting efficiency of the nanocomplex, thereby facilitating the internalization of FITC. The targeted uptake of pTSL@(P+I) by differentiated 3T3-L1 cells was mediated through specific interactions between peptides on the nanocomplex surface and prohibitin receptors on the cell surface.

### 3.5. pTSL@(P+I)-Induced Browning of White Adipocytes

The primary function of adipocytes is to store lipids in the form of triglycerides. Following the induction of differentiation and intervention as depicted in Figure 2C, the distribution and morphology of lipid droplets within adipocytes were assessed via Oil Red O staining. As illustrated in Figure 2D,E, the majority of adipocytes in the PBS group exhibited large single lipid droplets, which is a characteristic feature of white adipocytes. Following intervention with Piog, pTSL@P, pTSL@I, or pTSL@(P+I), varied reductions in the proportion of adipocytes containing large single lipid droplets were observed. Furthermore, an augmentation in the multilocularity of adipocytes was observed upon treatment with nanocomplexes, leading to the emergence of brown-adipose characteristics and a concomitant reduction in overall lipid droplet content. The quantitative analysis of lipid droplets extracted with isopropanol after Oil Red O staining is shown in Figure 2F. The optical density (OD) values of the Piog, pTSL@I, and pTSL@P treatment groups exhibited a sequential decrease in lipid content compared to that of the PBS group, accounting for 94.3%, 90%, and 85.7% of the PBS group, respectively. In addition, the OD values of the pTSL@(P+I)-treated group were significantly lower than those of the pTSL@P-treated (95.0%, *p* < 0.05) and pTSL@I-treated (90.5%, *p* < 0.001) groups. pTSL@P, pTSL@I, and pTSL@(P+I) clearly inhibited lipid droplet content, with pTSL@(P+I) exhibiting the strongest inhibitory effect. By quantifying the number of lipid droplets per cell in each group (Figure 2G), we observed that the pTSL@(P+I)-treated group exhibited the highest quantity of lipid droplets, and this disparity was found to be statistically significant when compared to that of the other groups.

To further confirm the lipolytic effect of pTSL@(P+I), we used a kit to quantify the levels of triglyceride and free fatty acid in adipocytes from different experimental groups. The results, as depicted in Figure 2H,I, demonstrated a sequential decrease in triglyceride content in the Piog, pTSL@I, and pTSL@P groups compared to that in the PBS group. Furthermore, the triglyceride content in the pTSL@(P+I) group was significantly lower than that in the pTSL@P and pTSL@I groups, accounting for 75% and 66%, respectively. As a decomposition product of triglyceride, the free fatty acid content in the pTSL@(P+I) group was significantly higher than that in the other four groups. These findings suggest that treatment with pTSL@(P+I) effectively reduced adipocyte triglyceride content, potentially through stimulation of hormone-sensitive lipase via PPARγ signaling. pTSL@P exhibited enhanced adipocyte penetration compared with free Piog, leading to improved lipid-lowering and pro-browning effects. Moreover, when combined with NIR light excitation, pTSL@I demonstrated the capability to effectively reduce adipocyte lipid levels. Furthermore, a synergistic pro-browning effect on adipocytes was observed for pTSL@(P+I) loaded with both Piog and IR780 under NIR light excitation, resulting in a more potent lipid-lowering effect than that of pTSL@P or pTSL@I.

Brown adipocytes possess a higher number of mitochondria and greater thermogenic activity than white adipocytes [48]. Therefore, measurement of mitochondrial activity serves as a crucial indicator of white adipose tissue browning. To assess this, we conducted mitochondrial fluorescence staining of adipocytes from each experimental group and the results are shown in Figure 2J,K. Based on the fluorescence intensity of CMXRos, the mitochondrial activity of adipocytes was significantly higher in the groups treated with pTSL@P and pTSL@(P+I) than in the PBS and Piog groups. Notably, the pTSL@(P+I)-intervention group exhibited the highest cellular fluorescence intensity. Interestingly, even in the pTSL@I-intervention group, there was a significant increase in cellular fluorescence intensity compared to that in the PBS group, indicating an enhancement in mitochondrial activity. These findings suggest that the photothermal effects of pTSL@I may induce mitochondrial biogenesis and white adipose tissue browning.

### 3.6. Molecular Mechanism of Pro-Browning by pTSL@(P+I) In Vitro

**Figure 3:**
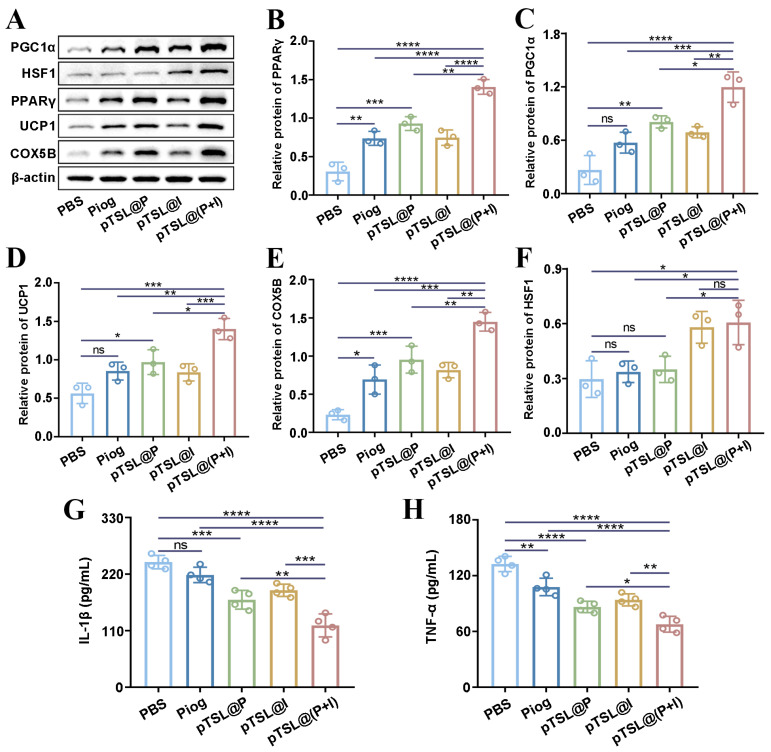
(**A**) Protein expression levels of PGC1α, HSF1, PPARγ, UCP1, and COX5B were assessed via Western blot analysis in each group of 3T3-L1 cells following induction of differentiation and intervention. (**B**–**F**) Quantitative analysis of the corresponding protein levels in (**A**) was conducted using Image J 1.8.0 software. The levels of inflammatory factors IL-1β (**G**) and TNF-α (**H**) were measured by ELISA in each group of 3T3-L1 cells following induction of differentiation and intervention. * *p* < 0.05, ** *p* < 0.01, *** *p* < 0.001, **** *p* < 0.0001; ns = not significant. Statistical significance was assessed via one-way ANOVA.

To further investigate the underlying molecular mechanisms, the protein expression levels of browning-related genes PPARγ, PGC1α, UCP1, and COX5B in 3T3-L1 cells were assessed via Western blotting. As shown in Figure 3A–E, compared to the PBS group with significantly lower protein expression, the intervention groups treated with Piog, pTSL@P, pTSL@I, and pTSL@(P+I) exhibited increased expression of these proteins. Moreover, the pTSL@(P+I) group showed a more pronounced increase in protein levels than the pTSL@P and pTSL@I groups, indicating synergistic pro-browning efficacy resulting from both the Piog pharmacological effect and the IR780 photothermal effect. The PPARγ/PGC1α transcription axis is a widely studied transcriptional pathway that regulates the process of white adipose tissue browning [49,50,51]. Piog, a PPARγ agonist, has the potential to induce white adipose tissue browning and enhance the expression of PGC1α and UCP1 [31]. Moreover, UCP1 is considered a crucial biomarker for the browning of white adipose tissue [19,52]. Further, COX5B is a functional protein involved in the respiratory chain that plays a crucial role in mitochondrial heat generation. As white adipose tissue underwent browning, the expression of COX5B also increased [53,54]. Furthermore, our observations revealed a significant upregulation of HSF1 protein expression in both the pTSL@I and pTSL@(P+I) groups compared to the other groups, with approximately twice the expression level in the PBS group (Figure 3F). HSF1 is a well-known heat-responsive protein, and previous research has demonstrated that localized heat therapy stimulates white adipose tissue browning by activating the HSF1/PGC1α transcriptional pathway [17,26,55]. Activation of this pathway has been linked to favorable metabolic outcomes, such as increased mitochondrial biogenesis and energy expenditure [25,26], resulting in improved obesity and related metabolic syndromes. The findings of this study provide evidence that NIR light energy absorbed by IR780 can be effectively converted into thermal energy for photothermal and pharmacological synergistic therapy through upregulation of UCP1 and COX5B expression via activation of the HSF1/PGC1α transcription axis.

Additionally, obesity is accompanied by heightened vascular permeability in adipose tissue and the presence of chronic inflammation, both of which are significant characteristics of this condition [56,57]. The levels of inflammatory cytokines IL-1β and TNF-α were assessed in cell culture supernatant of various intervention groups using ELISA. The results are presented in Figure 3G,H. Compared to the PBS group, the intervention treatments of Piog (IL-1β 89.8%, TNF-α 81.3%), pTSL@P (IL-1β 69.9%, TNF-α 65.2%), pTSL@I (IL-1β 77.5%, TNF-α 70.9%), or pTSL@(P+I) (IL-1β 49.5%, TNF-α 51.2%) led to varying degrees of suppression in IL-1β and TNF-α levels. Among the intervention groups, the pTSL@(P+I) group exhibited the most significant reduction in inflammatory cytokine levels, suggesting that pTSL@(P+I) can mitigate inflammatory responses and induce browning at the cellular level. We demonstrated that reduced inflammation was associated with improved obesity, consistent with previous findings highlighting the significance of anti-inflammatory approaches in managing obesity and related metabolic syndromes [58].

**Scheme 2:**
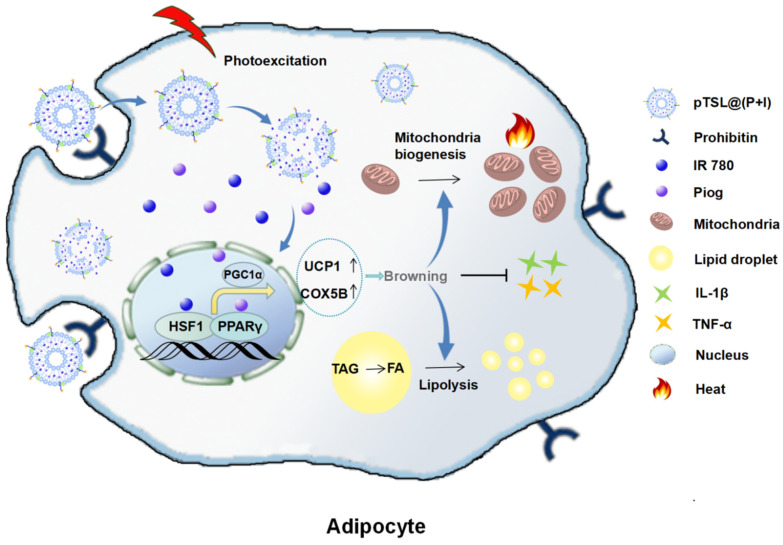
Anti-obesity mechanisms of pTSL@(P+I)-mediated photothermal and pharmacological synergistic therapy.

Based on the aforementioned results, we can tentatively infer the anti-obesity mechanism of pTSL@(P+I)-mediated photothermal and pharmacological synergistic therapy (Scheme 2). The targeted peptides facilitated efficient delivery of pTSL@(P+I) into adipocytes. Under the combined influence of photothermal therapy of IR780 and pharmacological therapy of Piog, there was an upregulation of UCP1 and COX5B expression by activating the transcription axis of HSF1/PGC1α and PPARγ/PGC1α, leading to a significant enhancement of white adipose tissue browning, increased energy expenditure, and reduced obesity.

### 3.7. Anti-Obesity Effect of pTSL@(P+I) on Diet-Induced Obesity Mice

**Figure 4:**
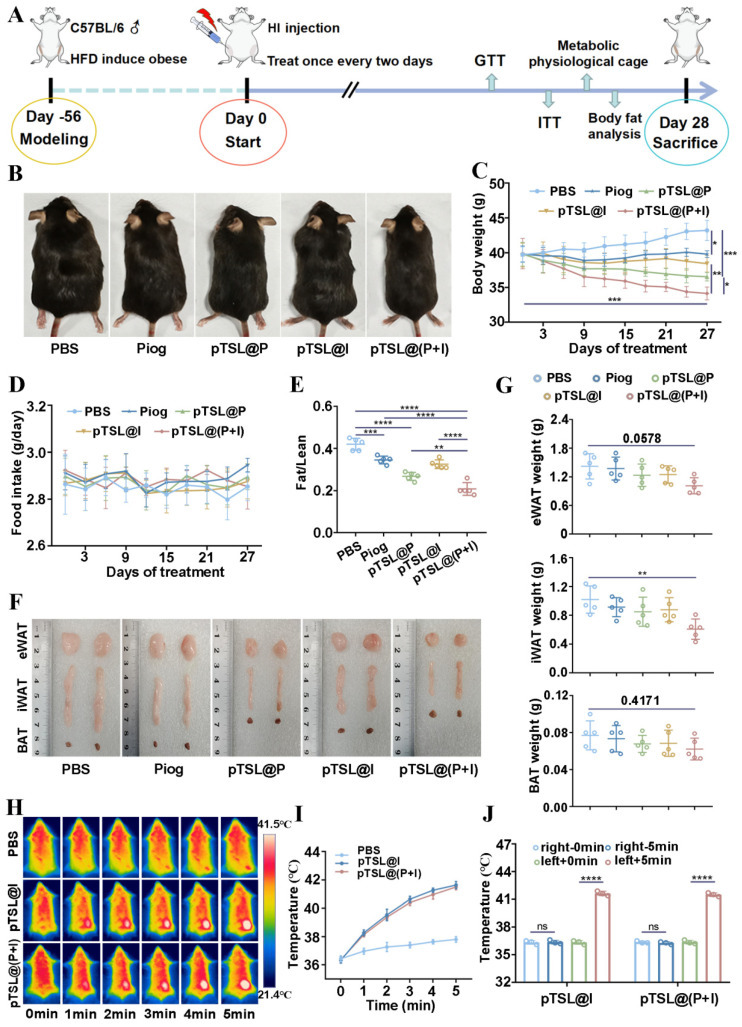
(**A**) A schematic diagram illustrating the in vivo experiments conducted on mice (HI: Latin for hypodermic injection). (**B**) Representative effect plots depicting the outcomes observed in each group of diet-induced obesity mice following a 4-week treatment. Assessment of daily changes in body weight (**C**) and dietary food intake (**D**) of mice (*n* = 5) in each intervention group. (**E**) Analysis of the weight ratio of fat/lean at the experiment’s endpoint using a small animal body fat analyzer (*n* = 5). Typical anatomical drawings (**F**) and corresponding weights (**G**) of eWAT, iWAT and BAT of mice (*n* = 5) in each experimental group at the endpoint. (**H**) After subcutaneous injection of nanocomplexes into both sides of the iWAT of mice in each group, infrared thermal imaging images of the left inguinal region of mice were irradiated by 808 nm NIR light (0.5 W/cm^2^) at different times. (**I**) Quantitative analysis of photothermal conversion in the left inguinal area of mice in the PBS, pTSL@I, and pTSL@(P+I) groups at different times with irradiation of 808 nm NIR light (0.5 W/cm^2^) in (H) (*n* = 3). (**J**) Quantitative analysis of left and right groin temperature before and after laser irradiation of left groin in pTSL@I and pTSL@(P+I) groups in (H) (*n* = 3). * *p* < 0.05, ** *p* < 0.01, *** *p* < 0.001, **** *p* < 0.0001; ns = not significant. Statistical significance was assessed via one-way ANOVA.

Subsequently, the targeted nanocomplex pTSL@(P+I) was applied to in vivo experimental studies to address obesity. Following a two-month period of HFD induction, we successfully established the diet-induced obesity mouse model (Supplementary Figure S2). The mice were randomly divided into five groups and administered bilateral subcutaneous injections of PBS, Piog, pTSL@P, pTSL@I, or pTSL@(P+I) into the iWAT (Figure 4A). After 28 days of treatment, mice in the intervention groups exhibited varying degrees of reduction in body size (Figure 4B) and weight loss (Figure 4C) compared with those in the PBS group. However, no significant differences in overall food intake were detected (Figure 4D) among the mice in this study. The weight of obese mice in the PBS group increased by 8.4% after 28 days, whereas various degrees of weight loss were observed in the other intervention groups throughout the treatment period. Notably, the pTSL@(P+I) group exhibited the greatest degree of weight loss, with a decrease of 14.4% compared to the initial weight. This weight loss was significantly greater than that observed in the Piog, pTSL@P, and pTSL@I groups, which experienced weight losses of 0.1%, 8.1%, and 3.1%, respectively. These findings suggested that the anti-obesity effect of pTSL@P was superior to that of free Piog, indicating that Piog loaded into pTSL@P exhibited more efficient weight loss. We consider that the weight loss caused by Piog may be related to its induced browning of white adipose, thereby increasing body energy expenditure. Interestingly, the combined pharmacological effects of Piog and photothermal effects of IR780 in pTSL@(P+I) resulted in a more pronounced anti-obesity effect than in either pTSL@P (*p* < 0.05) or pTSL@I (*p* < 0.01). This finding confirmed our hypothesis that this synergistic combination would result in enhanced weight loss.

Additionally, prior to reaching the experimental endpoint, we used a small animal body fat analyzer NMR Analyzer mq 7.5 to assess the fat and lean tissue weight ratios of the mice in each group (Figure 4E). The PBS group exhibited the highest fat/lean mass ratio of 41.8%, while the other groups demonstrated varying degrees of decrease in fat/lean mass ratios compared to the PBS group. Mice treated with pTSL@(P+I) displayed the lowest fat/lean mass ratio (20.8%), which was significantly lower than that in mice treated with Piog (34.8%), pTSL@P (26.9%), and pTSL@I (32.4%). These findings suggest that treatment with pTSL@(P+I) can effectively reduce body weight and body fat in obese mice.

Subsequently, we isolated and measured iWAT, eWAT, and BAT in each experimental group after euthanizing the mice (Figure 4F). Specifically, the pTSL@(P+I) group showed a reduction of approximately 40.4% and 28.7% in the masses of iWAT and eWAT, respectively, compared to the PBS group. However, no significant changes were observed in BAT volume or mass (Figure 4G). These results indicate that pTSL@(P+I) exerts potential anti-obesity effects by modulating the fat storage capacity of mice.

### 3.8. In Vivo Photothermal Characterization and Biodistribution of pTSL@(P+I)

To assess their in vivo photothermal effects, Piog, pTSL@P, pTSL@I, and pTSL@(P+I) were subcutaneously injected bilaterally into the iWAT of mice, with PBS serving as the control. Subsequently, 808 nm NIR light (0.5 W/cm^2^) was applied to irradiate the left inguinal area of the mice for 5 min. Infrared thermal imaging was conducted at 1 min intervals using an infrared thermal imager (FLIR E50) to monitor temperature variations in both inguinal areas. Notably, no significant alterations were observed in the temperature of the left inguinal region in the PBS, Piog, and pTSL@P groups over the entire 5 min. However, as shown in Figure 4H, under identical laser irradiation conditions, a substantial increase in temperature was observed in both pTSL@I and pTSL@(P+I) groups within the left inguinal region. Furthermore, with increasing duration of light exposure, there was a gradual rise in temperature, which eventually reached approximately 42 °C (Figure 4I). Additionally, quantitative analysis was performed to assess the temperatures in the left (+, with laser irradiation) and right (−, without laser irradiation) inguinal regions of the mice before (0 min) and after (5 min) laser irradiation in the pTSL@I and pTSL@(P+I) groups (Figure 4J). The results clearly indicate that laser irradiation significantly elevated the temperature of the left inguinal region in both groups. In contrast, no significant changes were observed in the temperature of the right side, which received only the nanocomplexes without laser irradiation. These findings indicated that IR780 retained its exceptional photothermal conversion efficiency in vivo. Additionally, subcutaneous injection of pTSL@(P+I) generated sufficient heat under NIR laser irradiation, thereby enabling a specific photothermal therapeutic effect. Simultaneously, it can achieve the phase transition temperature of the nanocomplex, facilitating the efficient release of Piog from pTSL@(P+I) and promoting the synergistic effect of photothermal therapy and pharmacological therapy for fighting obesity and related metabolic syndromes.

To investigate the in vivo biodistribution of pTSL@(P+I), FITC-labeled pTSL@(P+I) was subcutaneously injected into the left iWAT of mice. An intense fluorescence signal was observed at the injection site at 1 h post-injection. Over time, the fluorescence intensity gradually diminished but remained present within iWAT even after 48 h post-injection, indicating robust retention of the nanocomplex within this tissue (Supplementary Figure S3A). Mice were euthanized at both 24 and 48 h post-injection to collect various tissues for ex vivo imaging including iWAT, heart, liver, kidney, lung, and spleen. Notably, the strongest fluorescence signal from FITC-labeled pTSL@(P+I) was observed in iWAT at 24 h and weaker signals in the liver and kidneys. However, no fluorescent signal was observed in any organs other than iWAT after 48 h (Supplementary Figure S3B). These findings indicate that the nanocomplex exhibits a highly effective targeting capability for adipose tissue.

### 3.9. Physiological Effects of pTSL@(P+I) on Diet-Induced Obesity Mice

**Figure 5:**
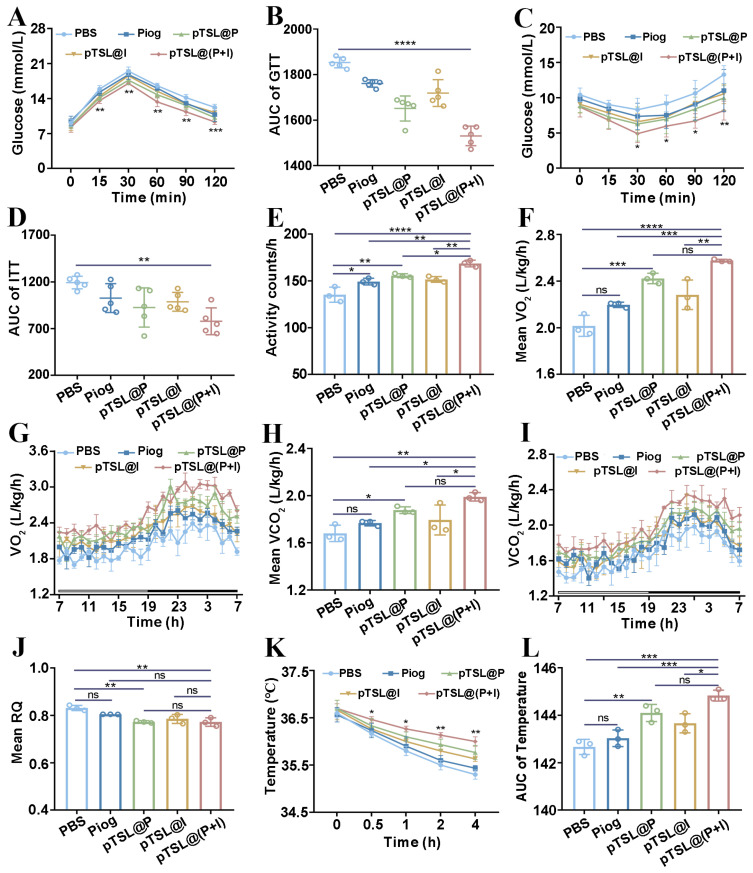
Glucose measurements and area under the curve of glucose tolerance test (GTT) (**A**,**B**) and insulin tolerance test (ITT) (**C**,**D**) in each group of diet-induced obesity mice after treatment (*n* = 5). The 24 h mean activity counts (**E**), mean and time-point O_2_ consumption (**F**,**G**), mean and time-point CO_2_ production (**H**,**I**), and mean respiratory quotient (RQ) (**J**) in mice analyzed by physiological metabolic cages (*n* = 3). Body temperatures of diet-induced obesity mice (**K**) and area under the curve (**L**) in each group during a 4 h cold tolerance test before the end of the experiment (*n* = 3). * *p* < 0.05, ** *p* < 0.01, *** *p* < 0.001, **** *p* < 0.0001; ns = not significant. Statistical significance was assessed via one-way ANOVA.

Subsequently, we conducted a comprehensive evaluation of the physiological impacts of pTSL@(P+I) intervention on diet-induced obesity mice using glucose tolerance tests (GTT) and insulin tolerance tests (ITT) prior to the conclusion of the experiment. The glucose tolerance test findings, depicted in Figure 5A,B, demonstrated that mice subjected to pTSL@(P+I) exhibited enhanced glucose tolerance compared with the PBS group (*p* < 0.0001), which was evident at almost all time points during the glucose tolerance test. Furthermore, mice treated with pTSL@(P+I) exhibited superior improvement in insulin sensitivity compared with the PBS group (*p* < 0.01), as indicated by the insulin tolerance test results (Figure 5C,D). These observations suggest that pTSL@(P+I) not only reduces body weight in mice, but also ameliorates the disrupted metabolic state.

A crucial determinant in combating obesity lies in maintaining a harmonious equilibrium between caloric intake and energy expenditure [59,60]. We conducted an analysis of 24 h O_2_ consumption, CO_2_ production, respiratory quotient (RQ), and physical activity counts in mice using metabolic physiology cages. As depicted in Figure 5E, the activity counts of mice were notably elevated in all other groups compared with the PBS group. Among them, the pTSL@(P+I) group displayed the highest activity counts, with a remarkable increase of 24.6% compared with the PBS group. Furthermore, the 24 h O_2_ consumption and CO_2_ production of mice in the Piog, pTSL@I, pTSL@P, and pTSL@(P+I) groups exhibited sequential increases compared to those of mice in the PBS group (Figure 5F–I), indicating that the metabolic rate of mice in the pTSL@(P+I) group was more vigorous than that of the other groups. Respiratory quotient, calculated as the ratio of released CO_2_ to consumed O_2_, was used to assess the fatty acid metabolism capacity, with a lower respiratory quotient ratio indicating a higher capacity for fatty acid metabolism [61]. The respiratory quotient of mice in the pTSL@(P+I) group was significantly lower than that of the other groups (Figure 5J), which were 7.2%, 4.1%, 0.2%, and 1.8% lower, respectively, when compared to the PBS, Piog, pTSL@P, and pTSL@I groups, suggesting that pTSL@(P+I) enhanced the lipid metabolism capacity in mice.

We also conducted a cold tolerance test to assess adaptive heat production in mice, which is a significant factor in energy expenditure in mice. During the 4 h exposure to cold, the body temperature of mice in the PBS group exhibited a significant decrease, whereas the temperature decline in the other groups was comparatively smaller. Notably, treatment with pTSL@(P+I) resulted in the lowest temperature decline in the diet-induced obesity mice. Compared with the PBS group, the pTSL@(P+I) group showed a statistically significant difference in temperature at every time point (*p* < 0.05), indicating that pTSL@(P+I) can effectively enhance the heat production capacity in mice (Figure 5K,L). Overall, these findings suggest that pTSL@(P+I) increases O_2_ consumption and potentially stimulates physical activity in mice. Application of pTSL@(P+I) may enhance fat and energy expenditure in diet-induced obesity mice, thereby promoting thermogenesis and weight loss.

**Figure 6:**
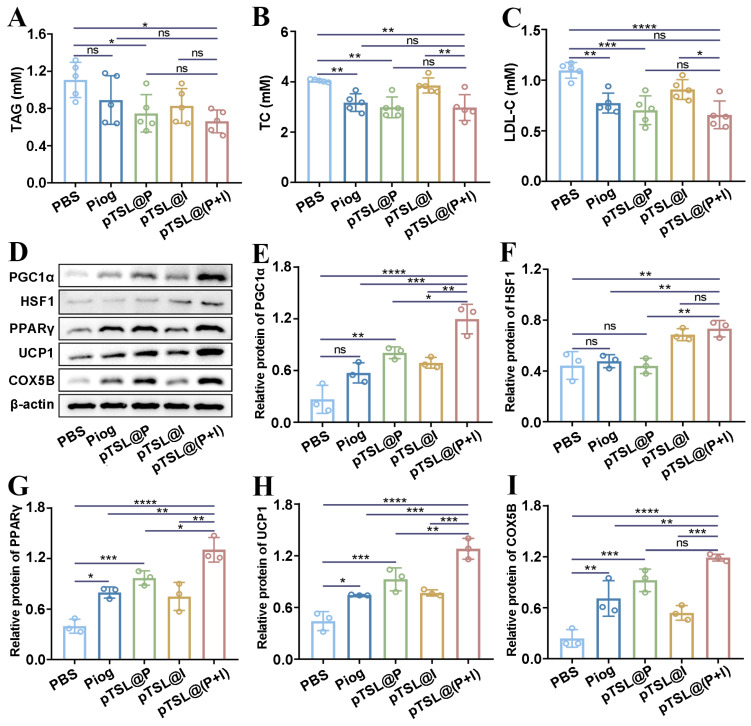
(**A**–**C**) Levels of lipid triglyceride (TAG), TC, and LDL-C in each group of mice (*n* = 5) at the endpoint of the experiment. Protein expression levels of PGC1α, HSF1, PPARγ, UCP1, and COX5B in iWAT after intervention were detected using Western blotting in each group of mice (**D**) and quantitative analysis of corresponding protein levels by Image J 1.8.0 software (**E**–**I**). * *p* < 0.05, ** *p* < 0.01, *** *p* < 0.001, **** *p* < 0.0001; ns = not significant. Statistical significance was assessed via one-way ANOVA.

Furthermore, compared to the PBS group, the lipid levels of mice in the other groups exhibited varying degrees of reduction (Figure 6A–C). Notably, the pTSL@(P+I) group demonstrated the most significant improvements in triglyceride, TC, and LDL-C levels. Compared to the PBS, Piog, pTSL@P, and pTSL@I groups, the triglyceride content decreased by 40.2%, 25.8%, 11.5%, and 20.0%, the TC content decreased by 26.4%, 6.2%, 0.3%, and 22.8%, and the LDL-C content decreased by 40.1%, 15.0%, 6.5%, and 27.5%, respectively, in the pTSL@(P+I)-intervention group. These findings suggest that the combined pharmacological and photothermal properties of pTSL@(P+I) can significantly decrease lipid levels and ameliorate obesity and related metabolic syndromes.

### 3.10. Mechanistic Study of pTSL@(P+I) Anti-Obesity Treatment

To investigate the potential relationship between weight loss in diet-induced obesity mice and white adipose tissue browning, proteins were extracted from iWAT after treatment. The protein expression levels of browning-related genes PPARγ, HSF1, PGC1α, UCP1, and COX5B were then determined using Western blot analysis. The results depicted in Figure 6D–I revealed that treatment with pTSL@(P+I) significantly upregulated the protein expression levels of UCP1 and PGC1α, surpassing 3-fold and 4-fold compared to the PBS group, respectively. Additionally, the expression level of COX5B, a marker of mitochondrial respiratory thermogenesis, was 5-fold higher than that in the PBS group. The protein expression of PPARγ in the intervention groups treated with Piog, in both the pTSL@P and pTSL@(P+I) groups, exhibited higher levels compared to the other groups, indicating that the nanocomplex-loaded Piog more effectively induced pro-browning effects mediated via activation of the PPARγ/PGC1α axis. Moreover, the expression of HSF1 in the intervention groups treated with IR780, in both the pTSL@I and pTSL@(P+I) groups, was significantly higher than that in the other groups. These findings are consistent with the above results in vitro as well as previous experimental conclusions [17,55], indicating that IR780 may further enhance activation of the HSF1/PGC1α axis through photothermal effects, thereby promoting white adipose tissue browning and exerting an anti-obesity effect.

**Figure 7:**
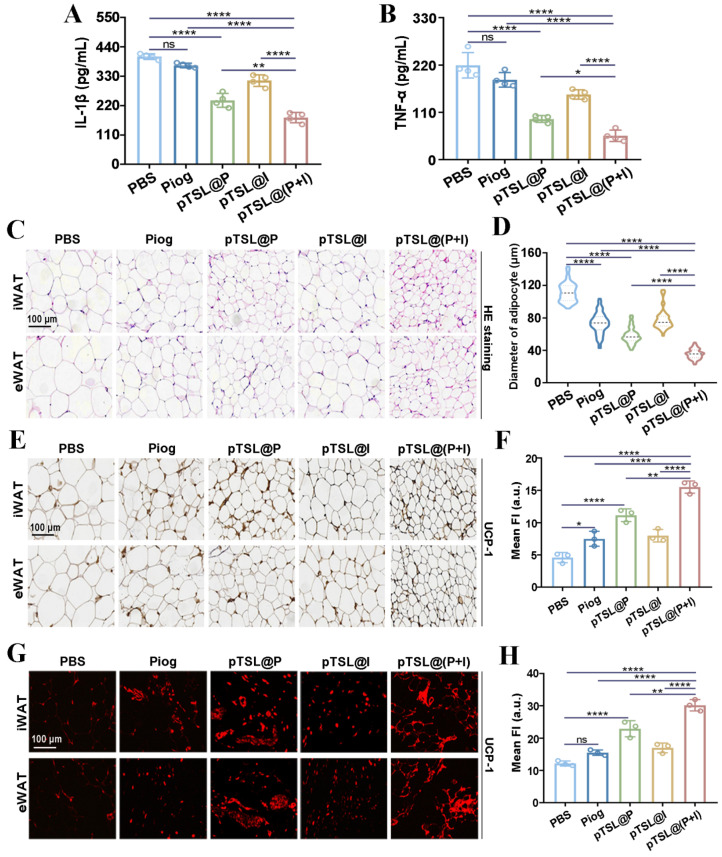
Levels of iWAT inflammatory cytokines IL-1β (**A**) and TNF-α (**B**) in mice measured by ELISA. (**C**) H&E staining of typical iWAT and eWAT of mice in each group after treatment. (**D**) Morphometric analysis of adipocyte cell sizes (*n* = 100 data points) in (**C**). Uncoupling protein 1 (UCP-1) labeled immunohistochemical staining (**E**) and quantification of UCP-1 expression (**F**) in (**E**) using Image J 1.8.0 software and UCP-1 labeled immunofluorescence images (**G**) and quantification of UCP-1 expression (**H**) in (**G**) using Image J 1.8.0 software of typical iWAT and eWAT of mice in each group after treatment. * *p* < 0.05, ** *p* < 0.01, **** *p* < 0.0001; ns = not significant. Statistical significance was assessed via one-way ANOVA.

To assess the potential correlation between weight loss in obese mice and an amelioration of inflammation, we collected iWAT tissues from each group and subjected them to ELISA analysis for quantification of IL-1β and TNF-α inflammatory factors. The results obtained are presented in Figure 7A,B. Notably, the PBS group exhibited the highest levels of IL-1β and TNF-α, whereas treatment with Piog, pTSL@P, pTSL@I, or pTSL@(P+I) led to varying degrees of suppression in these inflammatory cytokine levels. Notably, the pTSL@(P+I) group demonstrated a significant reduction in inflammatory cytokine levels by approximately 50% compared with the PBS group, suggesting that pTSL@(P+I) could effectively attenuate the inflammatory response while combating obesity in vivo.

To explore the additional pro-browning effects of pTSL@(P+I) beyond its molecular expression profiles, we examined the tissue structures of iWAT and eWAT in mice subjected to different interventions. Histological analysis using H&E staining (Figure 7C) revealed a reduction in the diameter of white adipocytes in the intervention group compared with that in the PBS group. Specifically, measurements of adipocyte diameter in iWAT demonstrated that the pTSL@(P+I) group exhibited the smallest adipocyte diameter, decreasing by 68.1%, 51.4%, 38.7%, and 54.3%, compared to those in the PBS, Piog, pTSL@P, and pTSL@I groups, respectively (Figure 7D). Therefore, we postulated that the observed weight loss resulting from the administration of pTSL@(P+I) may be attributed to the contraction of hypertrophic adipocytes in white adipose tissue.

Furthermore, our analysis of the immunohistochemistry and immunofluorescence data (Figure 7E–H) revealed that treatment with pTSL@(P+I) significantly upregulated the expression of uncoupling protein 1 compared to the other groups, particularly the pTSL@P and pTSL@I groups. These findings are consistent with the Western blot results, indicating that pTSL@(P+I) exhibits a superior ability to induce white adipose tissue browning, thermogenesis, and energy expenditure.

### 3.11. Safety Assessment of pTSL@(P+I)

To assess the safety of pTSL@(P+I), a comprehensive assessment of hepatic, renal, and cardiac functions was conducted in mice at the end of treatment, and the harvested organs were processed into H&E-stained tissue sections for histopathological examination (Supplementary Figure S4A). Additionally, an automated biochemical analyzer was used to measure the ALT, AST, TP, UA, SCr, UREA, CK, CKMB, and LDH levels (Supplementary Figure S4B–J). The results revealed no significant abnormalities compared with the PBS group, suggesting that pTSL@(P+I) was both safe and reliable.

## 4. Conclusions

In summary, we developed an adipocyte-targeted nanocomplex, pTSL@(P+I), with synergistic pharmacological and photothermal effects for promoting white adipose tissue browning and combating obesity through upregulating UCP1 and COX5B expression by activating the transcription axis of PPARγ/PGC1α and HSF1/PGC1α. This nanocomplex was locally injected subcutaneously into the adipose tissue. The targeted peptide facilitated efficient entry of the nanocomplex into adipocytes, whereas the photosensitizer induced a photothermal effect in subcutaneous adipocytes by NIR light excitation to control drug release. The combination of pharmacological and photothermal effects effectively promoted white adipose tissue browning and increased energy expenditure, ultimately leading to an improvement in obesity. This study suggests the suitability of utilizing the pTSL@(P+I) nanocomplex as a self-administered therapeutic intervention via subcutaneous injection, accompanied by brief application of NIR light in proximity to the target adipose tissue region. This unique ability to selectively target adipocytes and synergistically enhance browning is expected to translate into an innovative therapeutic approach to combat obesity and related metabolic syndromes.

## Data Availability

Data will be made available on request.

## Supplementary Materials

The following supporting information can be downloaded at: https://www.mdpi.com/article/10.3390/nano14161363/s1, Table S1: Characterization and properties of pTSL@(P+I), pTSL@P and pTSL@I. Figure S1: Dispersion coefficient stability of pTSL@(P+I) at 25 °C and 37 °C measured by DLS. Figure S2: Body weight of mice in control group and diet-induced obesity group. Figure S3: (A) In vivo fluorescence imaging showing the biodistribution of pTSL@(P+I) (labeled with FITC) at the indicated time points after direct subcutaneously injection into the left iWAT. (B) Ex vivo fluorescence imaging of different tissues at 24 and 48 h post-injection. Figure S4: (A) H&E staining of liver, kidney, and heart of mice in each group at the experimental endpoint; ALT, AST, TP (B–D) levels of liver function, UA, SCr, UREA (E–G) levels of kidney function and CK, CKMB, LDH (H–J) levels of heart function of mice in each group at the experimental endpoint.
