# Supplementary material for: Adipocyte-Targeted Nanocomplex with Synergistic Photothermal and Pharmacological Effects for Combating Obesity and Related Metabolic Syndromes
Source: Nanomaterials (Basel). 2024 Aug 19;14(16):1363. doi: 10.3390/nano14161363 (PMC11357138; doi:10.3390/nano14161363)
Supplement: Supplementary file 1 [file nanomaterials-14-01363-s001.zip › nanomaterials-3121916-supplementary.pdf]

Supplementary Materials

Table S1. Characterization and properties of pTSL@(P+I), pTSL@P and pTSL@I

Sample	D_H(nm)	PDI	Zeta Potential (mV)	EE (Piog) [%]	EE (IR780) [%]	LC (Piog) [%]	LC (IR780) [%]
pTSL@(P+I)1	191.20 ± 8.20	0.18 ± 0.01	-2.68 ± 0.69	57.20 ± 1.30	53.80 ± 0.30	9.20 ± 0.20	5.20 ± 0.40
pTSL@(P+I)2	200.30 ± 7.70	0.11 ± 0.04	-3.40 ± 0.43	53.90 ± 1.20	61.60 ± 0.70	10.10 ± 0.10	5.80 ± 0.30
pTSL@(P+I)3	211.70 ± 6.90	0.13 ± 0.03	-3.94 ± 0.54	52.40 ± 0.80	77.10 ± 0.40	11.00 ± 0.30	6.95 ± 0.60
pTSL@P	145.50 ± 0.70	0.12 ± 0.02	-3.16 ± 1.08	71.40 ± 0.70	--	13.70 ± 0.40	--
pTSL@I	224.70 ± 4.30	0.15 ± 0.02	-4.80 ± 1.27	--	65.20 ± 0.60	--	6.30 ± 0.50

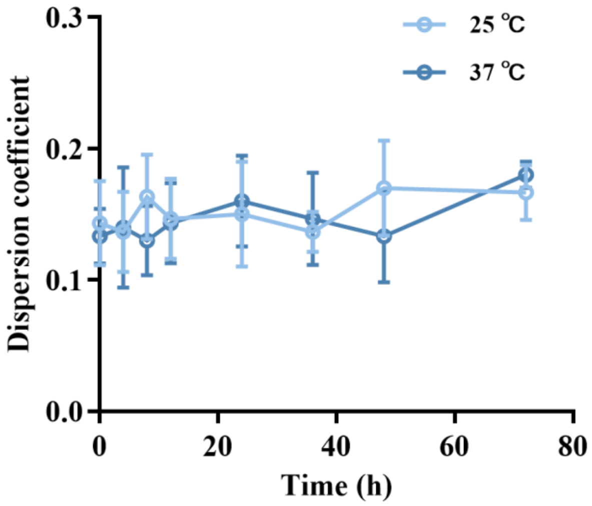

Figure S1. Dispersion coefficient stability of pTSL@(P+I) at 25 °C and 37 °C measured by DLS.

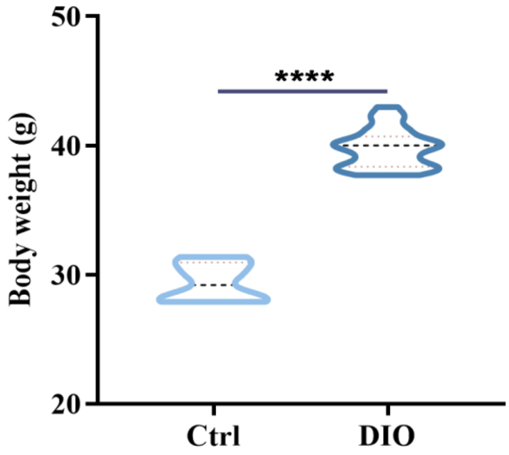

Figure S2. Body weight of mice in control group (n = 10) and DIO group (n = 30).

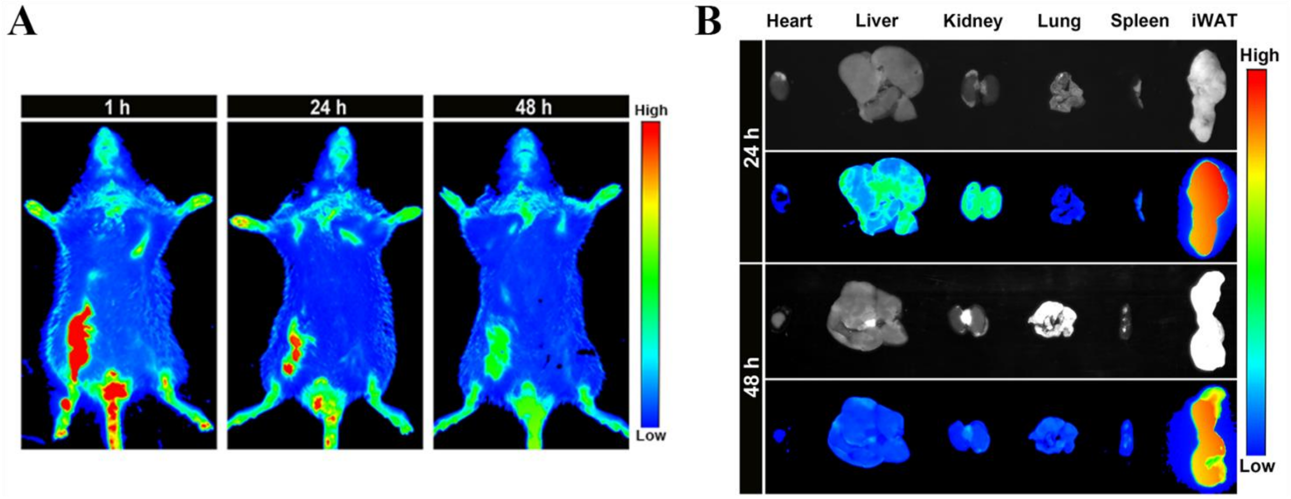

Figure S3. (**A**) *In vivo* fluorescence imaging showing the biodistribution of pTSL@(P+I) (labeled with FITC) at the indicated time points after direct subcutaneously injection into the left iWAT. (**B**) Ex vivo fluorescence imaging of different tissues at 24 and 48 h post-injection.

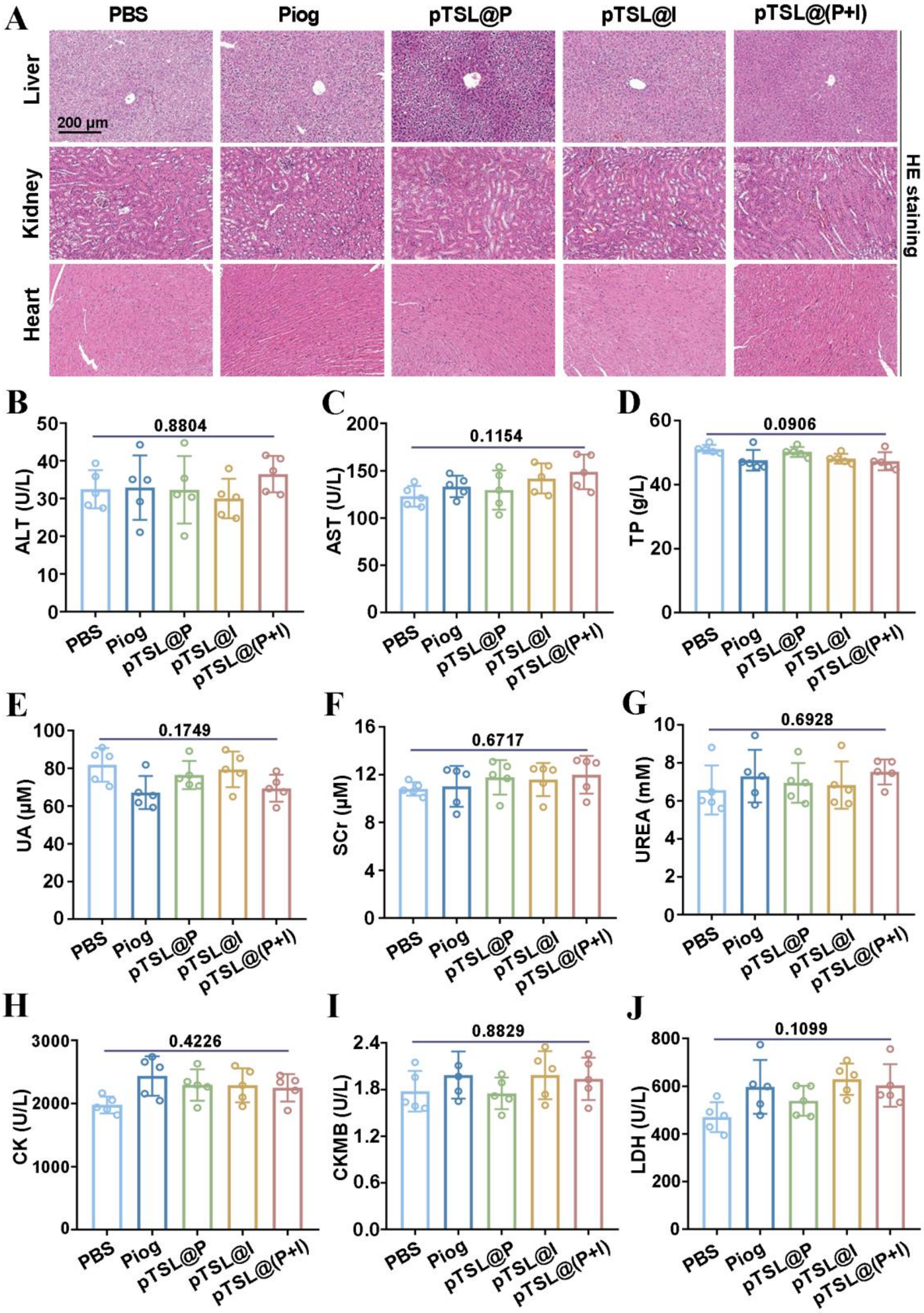

Figure S4. (**A**) H&E staining of liver, kidney and heart of mice in each group at the experimental endpoint. ALT, AST, TP (**B**-**D**) levels of liver function, UA, SCr, UREA (**E**-**G**) levels of kidney function and CK, CKMB, LDH (**H**-**J**) levels of heart function of mice (n = 5) in each group at the experimental endpoint.
